# Low Levels of GSTA1 Expression Are Required for Caco-2 Cell Proliferation

**DOI:** 10.1371/journal.pone.0051739

**Published:** 2012-12-10

**Authors:** Humaira Adnan, Holly Quach, Kimberley MacIntosh, Monica Antenos, Gordon M. Kirby

**Affiliations:** Department of Biomedical Sciences, University of Guelph, Ontario, Canada; National Institute of Agronomic Research, France

## Abstract

The colonic epithelium continuously regenerates with transitions through various cellular phases including proliferation, differentiation and cell death via apoptosis. Human colonic adenocarcinoma (Caco-2) cells in culture undergo spontaneous differentiation into mature enterocytes in association with progressive increases in expression of glutathione S-transferase alpha-1 (GSTA1). We hypothesize that GSTA1 plays a functional role in controlling proliferation, differentiation and apoptosis in Caco-2 cells. We demonstrate increased GSTA1 levels associated with decreased proliferation and increased expression of differentiation markers alkaline phosphatase, villin, dipeptidyl peptidase-4 and E-cadherin in postconfluent Caco-2 cells. Results of MTS assays, BrdU incorporation and flow cytometry indicate that forced expression of GSTA1 significantly reduces cellular proliferation and siRNA-mediated down-regulation of GSTA1 significantly increases cells in S-phase and associated cell proliferation. Sodium butyrate (NaB) at a concentration of 1 mM reduces Caco-2 cell proliferation, increases differentiation and increases GSTA1 activity 4-fold by 72 hours. In contrast, 10 mM NaB causes significant toxicity in preconfluent cells via apoptosis through caspase-3 activation with reduced GSTA1 activity. However, GSTA1 down-regulation by siRNA does not alter NaB-induced differentiation or apoptosis in Caco-2 cells. While 10 mM NaB causes GSTA1-JNK complex dissociation, phosphorylation of JNK is not altered. These findings suggest that GSTA1 levels may play a role in modulating enterocyte proliferation but do not influence differentiation or apoptosis.

## Introduction

The glutathione S-transferases (GSTs) are a multigene family of drug detoxification enzymes that are important in phase II metabolism by catalyzing the conjugation of glutathione to a variety of electrophilic substances [1]. GSTs are also involved in the biosynthesis and metabolism of prostaglandins [2], steroids [3] and leukotrienes [4], in the detoxification of products of lipid peroxidation [5] and in the acquisition of resistance to chemotherapeutic agents [6]. GST isoenzymes are also known to modulate cell signaling pathways that control cell proliferation and apoptotic cell death [7], [8] and have become potential therapeutic targets for the treatment of cancer [9].

Human colonic adenocarcinoma Caco-2 cells are extensively utilized as a model of intestinal epithelial cell differentiation as they have many phenotypic features of enterocytes [10]. *In vivo*, colonic epithelial cells are continuously renewing with a systematic turnover of cells involving transition between cell proliferation, differentiation and cell death by apoptosis. In culture, Caco-2 cells grow as an epithelial monolayer and undergo enterocyte-like differentiation with concomitant biochemical changes [11], [12]. Differentiation of Caco-2 cells is characterized by cell polarization, appearance of intercellular tight junctions and typical brush border microvilli projecting perpendicularly to the surface. The expression and activity of brush border enzymes notably alkaline phosphatase (AlkP), are increased in cellular differentiation [13]. Moreover, the expression of GSTA1 progressively increases as Caco-2 cells differentiate [14]. Terminally differentiated enterocytes undergo apoptosis and are sloughed from the surface epithelium into the intestinal lumen [15]. Therefore apoptosis seems to be a necessary component of colonocyte terminal differentiation. Indeed, neoplastic transformation of the colonic epithelium is associated with disordered regulation of cellular differentiation and apoptosis [16].

Numerous factors are involved in the control of intestinal epithelial cell differentiation, including growth factors, hormones, extracellular matrix proteins, vitamins, and luminal nutrients such as short chain fatty acids [17]. Sodium butyrate (NaB), a short-chain fatty acid present in the human large intestine, is derived from bacterial fermentation of complex carbohydrates. NaB is a preferred energy source for normal colonocytes *in vivo* but also reduces the growth and motility of colon cancer cell lines and causes dose-dependent cellular differentiation and apoptosis [18], [19], [20].

GSTs act as mediators of cell signaling kinase pathways involved in cell cycle transition such as proliferation and apoptosis [9]. Progressive increase in GSTA1 expression with cellular confluency in Caco-2 cells may influence responses to cellular stress [14]. Therefore we suspect that GSTA1 may function as a modulator of cell phase transitions. We have previously shown that the incidence of apoptosis stimulated by tumour necrosis factor α and sodium butyrate is significantly higher in preconfluent Caco-2 cells with minimal GSTA1 expression compared to differentiated postconfluent cells with high GSTA1 expression [14]. We have also demonstrated that GSTA1 forms complexes with c-Jun N-terminal kinase (JNK) and inhibits activation of JNK signalling in Caco-2 cells and that GSTA1 overexpression reduces phosphorylation of c-Jun during oxidative stress [14]. We hypothesized that low GSTA1 expression was a necessary condition for cell proliferation and that increased expression of GSTA1 is a requirement for Caco-2 cell differentiation. Our results indicate that low concentrations (1 mM) of NaB cause Caco-2 cell differentiation and concomitant GSTA1 induction and high concentrations (10 mM) stimulate apoptosis and down-regulation of GSTA1. Moreover, manipulation of GSTA1 levels by forced expression and knockdown using siRNA technology resulted in altered cell proliferation but did not affect NaB-mediated differentiation or sensitivity to apoptosis.

## Materials and Methods

### Materials

Dulbecco's modified eagle's medium (DMEM), fetal bovine serum (FBS), penicillin and streptomycin, mouse anti-β-actin antibody, reduced glutathione, para-nitrophenyl phosphate, para-nitrophenol and sodium butyrate were purchased from Sigma-Aldrich (Oakville, ON). The anti-GSTA1 rabbit antibody was purchased from Oxford Biomedical Research (Pickering, ON). Anti-GSTP1 rabbit antibody was purchased from Biotrin (Mississauga, ON). Mouse anti-V5 antibodies, TryPLE Express, Stealth™ siRNA sequences and pcDNA 3.1/V5-His TOPO TA Expression Kit, TRIzol reagent, MMLV (Moloney-murine-leukaemia virus) RT were purchased from Invitrogen (Burlington, ON). 5-Androstene-3,17-dione was purchased from Steraloids Inc (Newport, RI). An ECL Plus kit was purchased from GE Health Sciences (Oakville, ON). A DNA Master SYBR Green I kit and 5-bromo-2-deoxyuridine (BrdU) chemiluminescent enzyme-linked immunosorbant assay (ELISA) kit was purchased from Roche Diagnostics (Mississauga, ON, Canada). CytoTox-ONE Assay kits and Aqueous Non-Radioactive Cell Proliferation (MTS) Assay kits were purchased from Promega (Whitby, ON). A SAPK/JNK Kinase assay kit, and rabbit anti-caspase-3 antibodies were purchased from Cell Signaling Technology, Inc (Pickering, Ontario).

### Cell culture and treatments

Human adenocarcinoma (Caco-2) cells, obtained from the ATCC (Cedarlane Inc., Burlington, ON) were cultured in DMEM supplemented with 10% (v/v) FBS and 100 µg/mL of penicillin and streptomycin in 5% CO_2_ at 37°C. All experiments utilized preconfluent cells with 80% confluency and 10 d postconfluent Caco-2 cells between passages 8–40. Wild type Caco-2 and GSTA1-modulated Caco-2 cells were treated with sodium butyrate (NaB, 1 mM and 10 mM) in serum-free media for 24 to 72 h.

### RNA isolation and real-time RT–PCR analysis

Total RNA was isolated using TRIzol reagent, according to the manufacturer's instructions. Isolated RNA (one μg/20 μl of reaction volume) and one unit of RQ1 DNAse were used to prepare DNase-treated RNA. Complementary DNA (cDNA) was then generated from the DNase-treated RNA using 0.1 μg of random primers, 20 units of RNase inhibitor, 10 mM dNTPs (Invitrogen) and 200 units of MMLV (Moloney-murine-leukaemia virus) RT. Real-time PCR was performed on a Roche Light Cycler using a DNA Master SYBR Green I kit. The PCR reaction was done in a volume of 10 μl, containing 1 μl of SYBR Green I, 5 μM of each primer and 2 mM MgCl_2_. Oligonucleotide primers for the differentiation makers AlkP were 5′ CTCCAACATGGACATTGACG and 3′ TGAGATGGGTCACAGACTGG, villin were 5′ AGCCAGATCACTGCTGAGGT and 3′ TGGACAGGTGTTCCTCCTTC, dippeptidyl peptidase-4 (DDP-4) were 5′ GGCGTGTTCAAGTGTGGAAT and 3′ TCTTCTGGAGTTGGGAGACC, E-cadherin were 5′ TGATCGGTTACCGTGATCAAAA and 3′ GTCATCCAACGGGAATGCA and for glyceraldehyde-3-phosphate dehydrogenase (GAPDH) were 5′ CAGTCCATGCCATCACTGCC and 3′ GCCTGCTTCACCACCTTCTTG. The PCR parameters were 95°C for 1 min, 1 cycle, and 35 cycles of 95°C for 15 s, 70°C for 5 s and 72°C for 15 s. Messenger RNA levels for these differentiation markers were normalized against GAPDH mRNA.

### Transient transfections

For transfections, Caco-2 cells (10^6^ cells per 6 cm^2^ dish) were transiently transfected in suspension using Lipofectamine 2000 as recommended by the manufacturer. Preconfluent cells were transfected with non-specific negative control siRNA (NS siRNA) or GSTA1-specific siRNA (GST siRNA). The sequences of GSTA1 siRNA were 20: 5′ AAGACUGGAGUCAAGCUCCUCGACG and 3′ CGUCGAGGAGCUUGACUCCAGUCUU, GSTA1 siRNA 19: 5′ AGUUCCACCAGAUGAAUGUCAGCCC and 3′ GGGCUGACAUUCAUCUGGUGGAACU and GSTA1 siRNA 18: 5′ UGGACAUACGGGCAGAAGGAGGAUC and 3′ GAUCCUCCUUCUGCCCGUAUGUCCA. Transfections were performed in Opti-MEM using a final concentration of 40 nM of siRNA for 72 h. GSTA1 protein and enzymatic activity were assessed by western blotting and 5-androstene-3,17-dione (AD) assay respectively to confirm GSTA1 specific silencing.

A GSTA1 expression vector was constructed. Briefly, the cDNA for GSTA1 was cloned in pcDNA 3.1/V5-His TOPO using primers 5′ AAACCTGAAAATCTTCCTTGCTTCTT and 3′ GAAACCTCCAGGAGACTGCTA. Plasmid inserts were confirmed by DNA sequence analysis at the University of Guelph, Laboratory Services Division. One µg/mL of GSTA1 pcDNA 3.1/V5-His TOPO plasmid was used in all experiments. Empty vector (pcDNA 3.1) was used as a transfection control. Caco-2 cells (10^6^ cells per 6 cm^2^ dish) were transfected in suspension to over-express GSTA1 protein. Protein levels of GSTA1-V5 were assessed by immunoblotting procedures using mouse anti-V5 antibody to confirm over-expression.

### GSTA1 activity assay

Cells were harvested with 50 mM Tris HCl pH 8.0 and 0.1 mM EDTA followed by sonication on ice and centrifugation at 9000 g, 4°C for 20 min. GSTA1 activity (nmol/mg/min) was assessed using 10 mM AD as substrate (dissolved in 100% methanol) in the presence of 50 mM glutathione-reduced (dissolved in 50 mM Tris pH 8) and 200 µg of protein at 248 nm at 30°C [21]. All the reagents were prepared fresh and kept on ice.

### Alkaline phosphatase (AlkP) activity assay

To determine AlkP activity, cells were harvested in PBS and whole cell lysates (400 µg of protein) were used in the reaction mixture. AlkP activity (µmol/mg/min) was determined using para-nitrophenyl phosphate as substrate as previously described [22]. The alkaline phosphatase activity in each sample was calculated from a para-nitrophenol standard curve.

### Cytotoxicity assay

Cytotoxicity was determined by lactate dehydrogenase (LDH) release using a CytoTox-ONE™ kit (Promega) according to the manufacturer's protocol. Caco-2 cells (15,000 cells per well) were plated in 96-well clear bottom black plates. Cells were treated with NaB (1 mM and 10 mM) for 24 h to 72 h. LDH release was measured at 544/590 nm using a FluoStar OPTIMA fluorimeter (BMG Labtech). Percent cytotoxicity was calculated by subtracting the background and dividing the LDH release from control wells as recommended by the manufacturer.

### MTS assay

Cellular proliferation was determined using a CellTiter 96 Aqueous Non-Radioactive Cell Proliferation Assay MTS kit (Promega) according to the manufacturer's instruction. Caco-2 cells (15,000 cells per well) were plated in 96-well plates. 20 µl of the MTS reagent was added to each well containing 100 µl culture medium. The plate was incubated for 2 h at 37°C in a humidified, 5% CO_2_ atmosphere. The plate was then read at 490 nm using a plate reader.

### BrdU Incorporation

Cell proliferation was assessed by determining BrdU incorporation using the 5-bromo-2-deoxyuridine (BrdU) chemiluminescent enzyme-linked immunosorbant assay (ELISA) kit (Roche). Caco-2 cells were incubated with a 10 µM BrdU solution for 2 hours at 37°C, then fixed and denatured and incubated with a peroxidase-conjugated antibody against BrdU (1∶100) for 1 hour at room temperature. The cells were then incubated with the hydrogen peroxide substrate solution and fluorescence was read at 460 nm using a Fluostar OPTIMA fluorimeter (BMG Labtech).

### Flow cytometry

Caco-2 cells (10^6^ cells per 6 cm^2^ dish) were plated for the assay, and flow cytometry analysis was done as described previously [23]. Before harvesting, the cells were washed twice with 1×PBS, followed by centrifugation at 6000 rpm for 10 min. The pellet was resuspended with 70% ethanol for 30 min at 4°C. Cells were again centrifuged at 6000 rpm for 10 min and were incubated with the DNA-binding dye propidium iodide (PI) solution (containing 0.1% sodium citrate, 0.1% Triton X-100, and 50 mg/L PI in water) for 1 hour at room temperature. Cells were then analyzed using a FACS (Fluorescence-activated cell sorting) caliber flow cytometer (BD FACSCalibur, Becton, Dickinson and Company Biosciences, San José, USA).

### Assessment of GSTA1-JNK complexes

GSTA1-JNK complexes were detected from cells using c-Jun fusion protein beads from the SAPK/JNK Kinase assay kit (Cell Signaling Technologies Inc.). Sample protein (350 µg) was incubated with 10 µl of c-Jun fusion protein beads with gentle rocking overnight at 4°C to pull-down JNK. Samples were centrifuged at 14000 g for 2 min at 4°C. The pellet was washed 4 times with lysis buffer and the immunoprecipitates were resuspended with 3X SDS buffer containing 150 mM DTT. Samples were boiled at 95°C for 5 minutes to denature the proteins and subject to SDS-PAGE.

### SDS-PAGE and Western blot analysis

Caspase-3, p-JNK and GSTA1 and GSTP1 expression were assessed by western blot analysis. Cells were harvested with lysis buffer and stored at −80°C. The cell extracts were then thawed and sonicated on ice for 10 minutes and centrifuged at 9000 rpm for 20 min at 4°C. Protein was quantified by the Bradford assay using BSA as a standard. Protein (30 µg) was separated by 12% SDS/PAGE and transferred to nitrocellulose membranes that were blocked in 5% milk in Tris-buffered saline with 0.1% Tween (TBS/T) and incubated overnight with either rabbit monoclonal anti-Caspase-3 (1∶1000) antibody; rabbit polyclonal anti-GSTA1 (1∶5000) antibody: rabbit polyclonal anti-GSTP1 antibody (1∶500); or rabbit monoclonal anti p-JNK (1∶1000) antibody. After 1 h of incubation with a horseradish peroxidase-conjugated anti-rabbit antibody, bands were detected by chemiluminescence (ECL Plus) and visualized using a Typhoon 9410 scanner (GE Health Care). The densitometric analysis of protein was determined using Image J (NIH software). β-actin was detected as a protein loading control.

### Statistical analysis

One-way ANOVA and two-way ANOVA test were used to assess statistically significant differences among treatment groups for the analysis of single variance and multiple variances respectively. For single variance, the Tukey's least significant test was used for comparison and for multiple variances Bonferroni's hoc test was applied. Significance was established at p values <0.05.

## Results

### GSTA1 levels increase in differentiating Caco-2 cells

**Figure 1:**
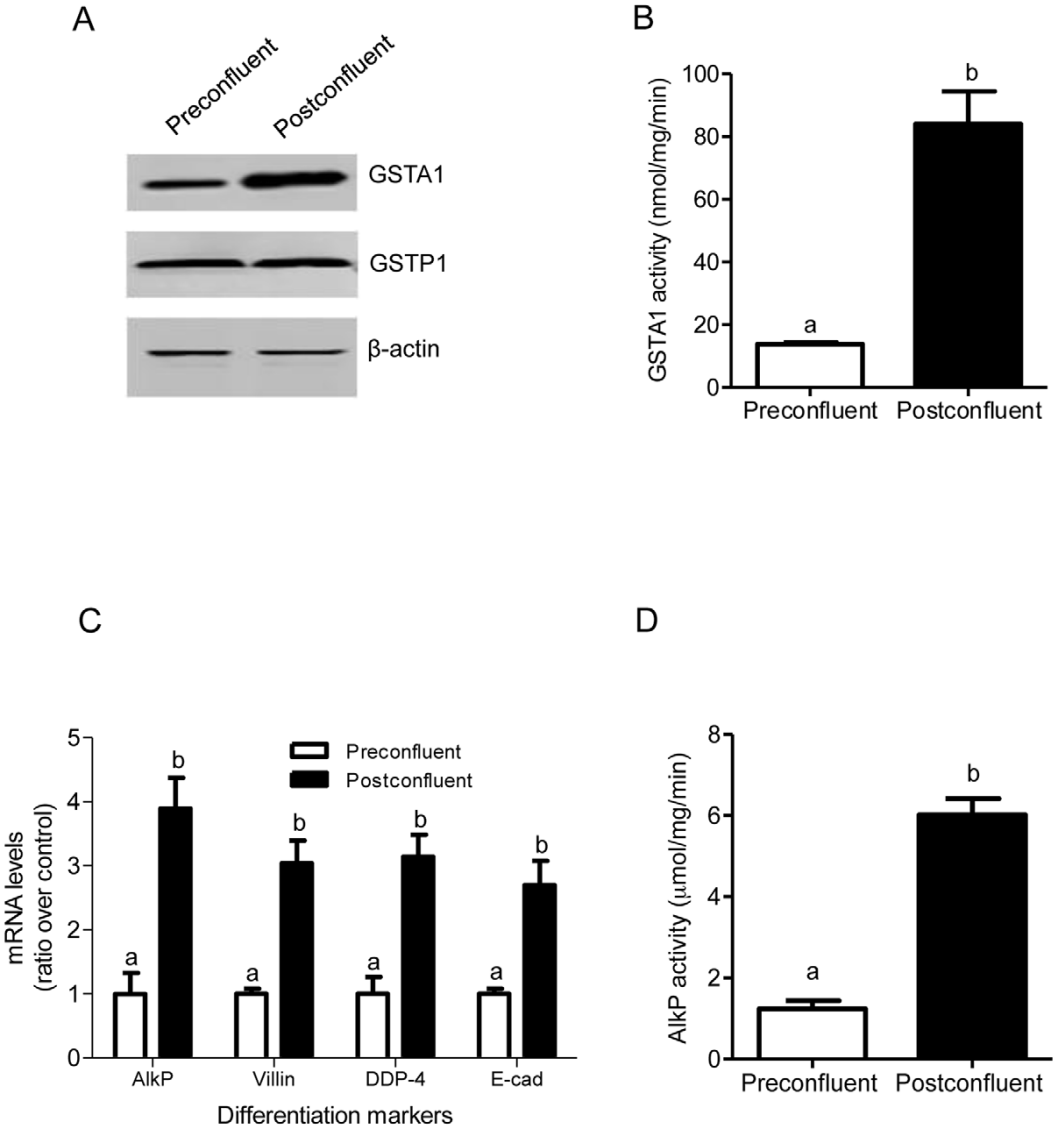
GSTA1 levels increase in differentiating Caco-2 cells. Preconfluent and 10 d postconfluent Caco-2 cells were assessed for: (A) protein expression of GSTA1 (∼25 KDa) and GSTP1 (∼26 KDa). β-actin was used as a protein loading control; (B) GSTA1 enzyme activity (nmol/mg/min); (C) mRNA levels of differentiation markers: AlkP, villin, DPP-4 and E-cadherin by real time RT-PCR; and (D) AlkP enzyme activity (µmol/mg/min). Values represent the mean ± S.E. of three independent experiments with three replicates each. Bars indicated by different letters differ significantly from one another (p≤0.001).

Caco-2 cells in culture progressively undergo spontaneous enterocytic differentiation with increasing days of postconfluency. We have previously shown that GSTA1 protein expression increases with confluency in Caco-2 cells [14]. To investigate the relationship between GST expression and Caco-2 cell differentiation we compared GSTA1 and GSTP1 protein levels in preconfluent and postconfluent Caco-2 cells. While GSTA1 protein levels significantly increased in 10 d postconfluent cells, GSTP1 expression did not change (Fig. 1A). Moreover, GSTA1 activity increased 6.1-fold (p<0.001) from 13.8 nmol/mg/min in preconfluent cells to 84.1 nmol/mg/min in 10 d postconfluent cells (p<0.001) (Fig. 1B).

We verified that specific genes associated with cellular differentiation were transcriptionally up-regulated by examining the mRNA transcript levels of various differentiation markers. The mRNA levels of alkaline phosphatase (AlkP), villin, dipeptidyl peptidase-4 and E-cadherin in preconfluent and 10 d postconfluent Caco-2 cells were assessed by real time RT-PCR (Fig. 1C). Transcripts of AlkP, villin, DDP-4 and E-cadherin significantly increased by 3.9- (p<0.001), 3.1- (p<0.001), 3.1- (p<0.001) and 2.7- (p<0.01) fold, respectively, in postconfluent cells (Fig. 1C). Moreover, a five-fold change in AlkP activity from 1.2 µmol/mg/min in preconfluent cells to 6.0 µmol/mg/min in 10 d postconfluent cells was observed (p<0.05) (Fig. 1D).

### Modulation of GSTA1 levels alters proliferation of Caco-2 cells

**Figure 2:**
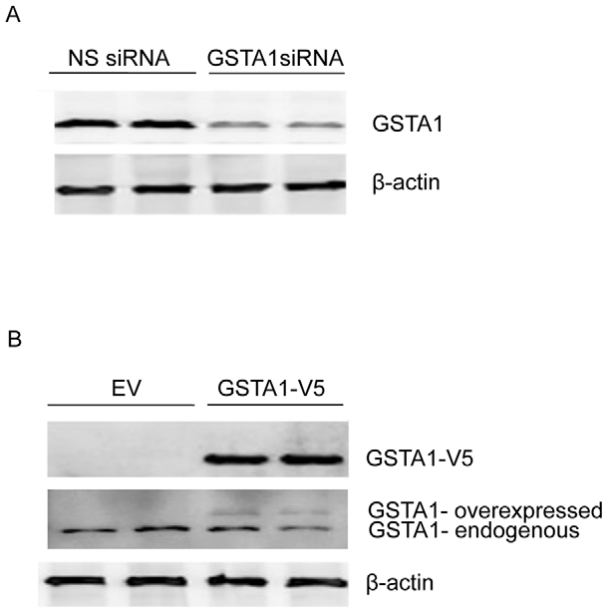
GSTA1 levels can be modulated in preconfluent Caco-2 cells. (A) Representative western blot of GSTA1 (∼25 KDa) protein expression in preconfluent Caco-2 cells that were transiently transfected with 40 nM of GSTA1 siRNA or non-specific (NS) siRNA for 72 h. (B) Representative western blot of V5 (∼26 KDa) protein expression in preconfluent Caco-2 cells that were transiently transfected with one µg of GSTA1-V5 or empty vector (EV) for 48 h. β-actin (∼42 KDa) was used as a protein loading control in all panels.

Since GSTA1 levels progressively increase with confluency in differentiating Caco-2 cells, we investigated the relationship between GSTA1 and cellular proliferation. For this purpose, we transiently modulated GSTA1 expression levels in preconfluent cells and confirmed GSTA1 down-regulation or over-expression by western blot analysis and enzyme activity (Figure 2 and Table 1). Preconfluent cells were transiently transfected with GSTA1 siRNA and non-specific negative control (NS) siRNA for 72 h to down-regulate GSTA1 (Fig. 2A). The protein levels significantly decreased by 68% (p<0.001) in GSTA1 siRNA-transfected cells as compared to controls (Fig. 2A and Table 1). Preconfluent cells were transiently transfected with a GSTA1-V5 expression plasmid and empty vector (EV) for 48 h to overexpress GSTA1. Western blot analysis of transfected cells using an anti-V5 antibody confirmed that expression of GSTA1-V5 occurred only in GSTA1-V5-ransfected cells and was absent in EV-transfected cells (Fig. 2B). In cells transiently transfected with GSTA1-V5, total GSTA1 activity increased 3.5-fold (p<0.001) from 2.8 nmol/mg/min in cells transfected with EV to 9.9 nmol/mg/min (Table 1).

**Table 1:** Relative abundance and activity of GSTA1 in transiently transfected Caco-2 cells.

	GSTA1 protein levels*	GSTA1 activity* (nmol/mg/min)
***GSTA1 silencing***		
GSTA1 siRNA	0.31±0.02 ^b^	2.10±0.16 ^b^
NS siRNA	1±0.12 ^a^	4.50±0.31 ^a^
***GSTA1 overexpression***		
GSTA1-V5	1.20±0.05 ^b^	9.90±0.42 ^b^
empty vector	ND	2.80±0.93 ^a^

* Each value represents the mean ± S.E of three independent experiments with three replicates each. ND, not determined; NS, non-specific. Values with different letters are significantly different from each other.

**Figure 3:**
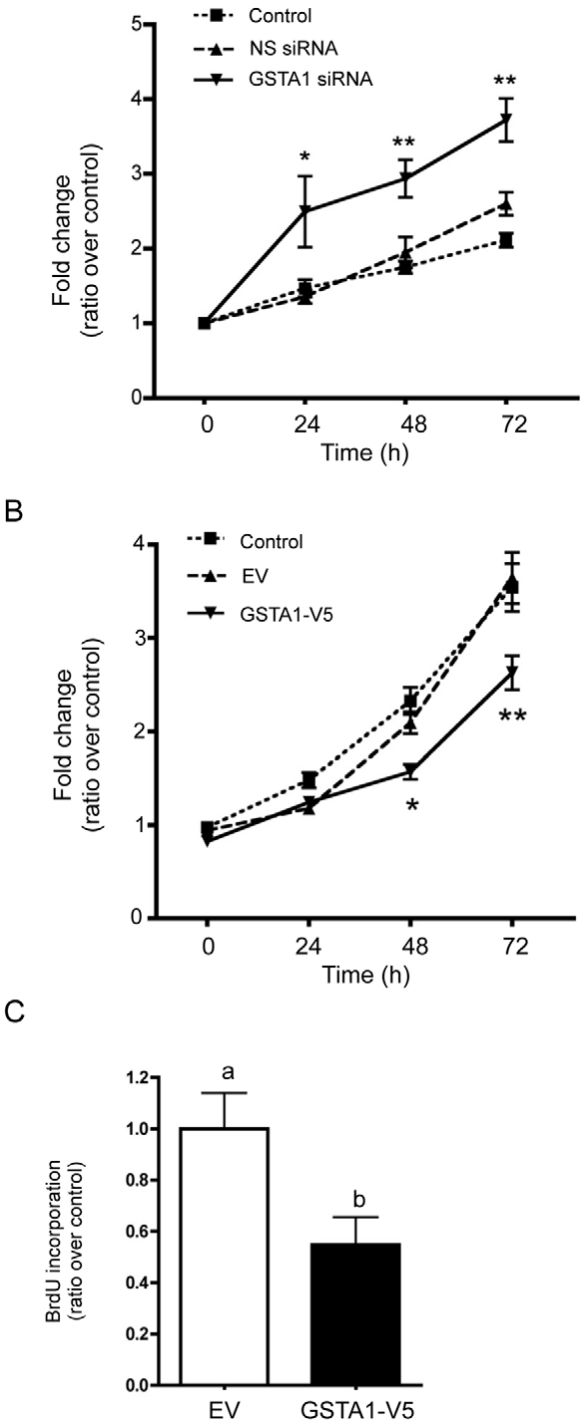
Modulation of GSTA1 levels mediate changes in Caco-2 cell growth. Effect of (A) GSTA1 down-regulation and (B) GSTA1-V5 overexpression on Caco-2 cell viability evaluated by MTS assay over three days. Asterisks depict significant differences between controls and the cells with GSTA1 modulated levels (*, *p*≤0.05; and **, *p*≤0.01). (C) Effect of GSTA1-V5 over-expression on cellular proliferation at 72 h as determined by BrdU incorporation in Caco-2 cells. Bars indicated by different letters differ significantly from one another (p≤0.001). Values represent the mean ± S.E. of four independent experiments with three replicates each.

To examine the effect of GSTA1 knockdown or over-expression on cellular proliferation, a MTS assay was performed for up to 72 h (Fig. 3A and B). GSTA1 knockdown significantly increased cell proliferation at 24 (p<0.05), 48 (p<0.01) and 72 h (p<0.01) as compared to controls (Fig. 3A). In Caco-2 cells overexpressing GSTA1, a significant decrease in proliferation at 48 h (p<0.05) and 72 h (p<0.01) was observed when compared to controls (Fig. 3B). Similar results were obtained when cells were labeled using bromodeoxyuridine (BrdU). BrdU incorporation decreased significantly to 54% of control levels in cells overexpressing GSTA1 (Fig. 3C). No significant increase in cytotoxicity was observed due to transfections in GSTA1 knock-down or overexpressed Caco-2 cells (data not shown).

### GSTA1 down-regulation affects cell cycle progression

**Figure 4:**
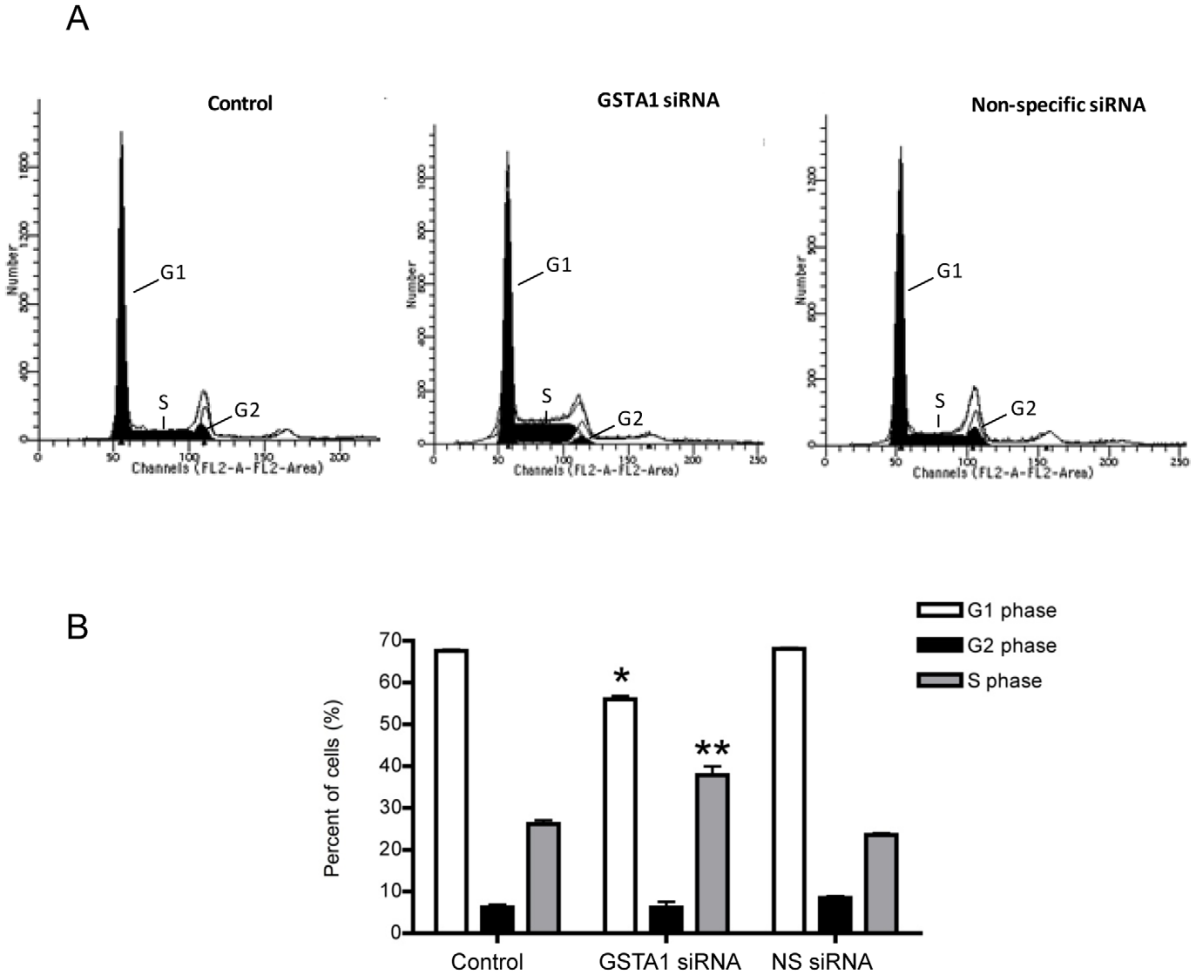
GSTA1 down-regulation increases the percentage of Caco-2 cells in the S phase. (A) Changes of cell cycle phase distribution in GSTA1 down-regulated Caco-2 cells as compared to controls. (B) Graphic representation of percent of cells in G1, S and G2 phase of cell cycle in non-transfected control, GSTA1 siRNA and NS siRNA transfected Caco-2 cells. Asterisks depict significant differences between control and GSTA1 down-regulated cells (*, p≤0.05; and **, p≤0.01).

We analysed the changes of cell cycle phase distribution in GSTA1 down-regulated Caco-2 cells. Forty-eight hours after transfection with GSTA1 siRNA, FACS analysis was performed to assess the influence of GSTA1 knockdown on cell cycle progression. While Caco-2 cells transfected with non-specific siRNA had a cell cycle distribution identical to untransfected control cells (Fig. 4A), the distribution of cell cycle phases was significantly altered in cells in which GSTA1 was knocked down. Fifty-five percent of cells transfected with GSTA1 siRNA were in G1 phase of the cell cycle compared 68% in controls and cells transfected with non-specific siRNA (p<0.05) (Figure 4A and 4B). In addition, over 42% of the cells accumulate in the S phase of the cell cycle with GSTA1 knockdown (p<0.01) compared to 24% in controls and cells transfected with non-specific siRNA (Fig. 4B).

### GSTA1 activity is altered with NaB-mediated changes in cell cycle phase

**Figure 5:**
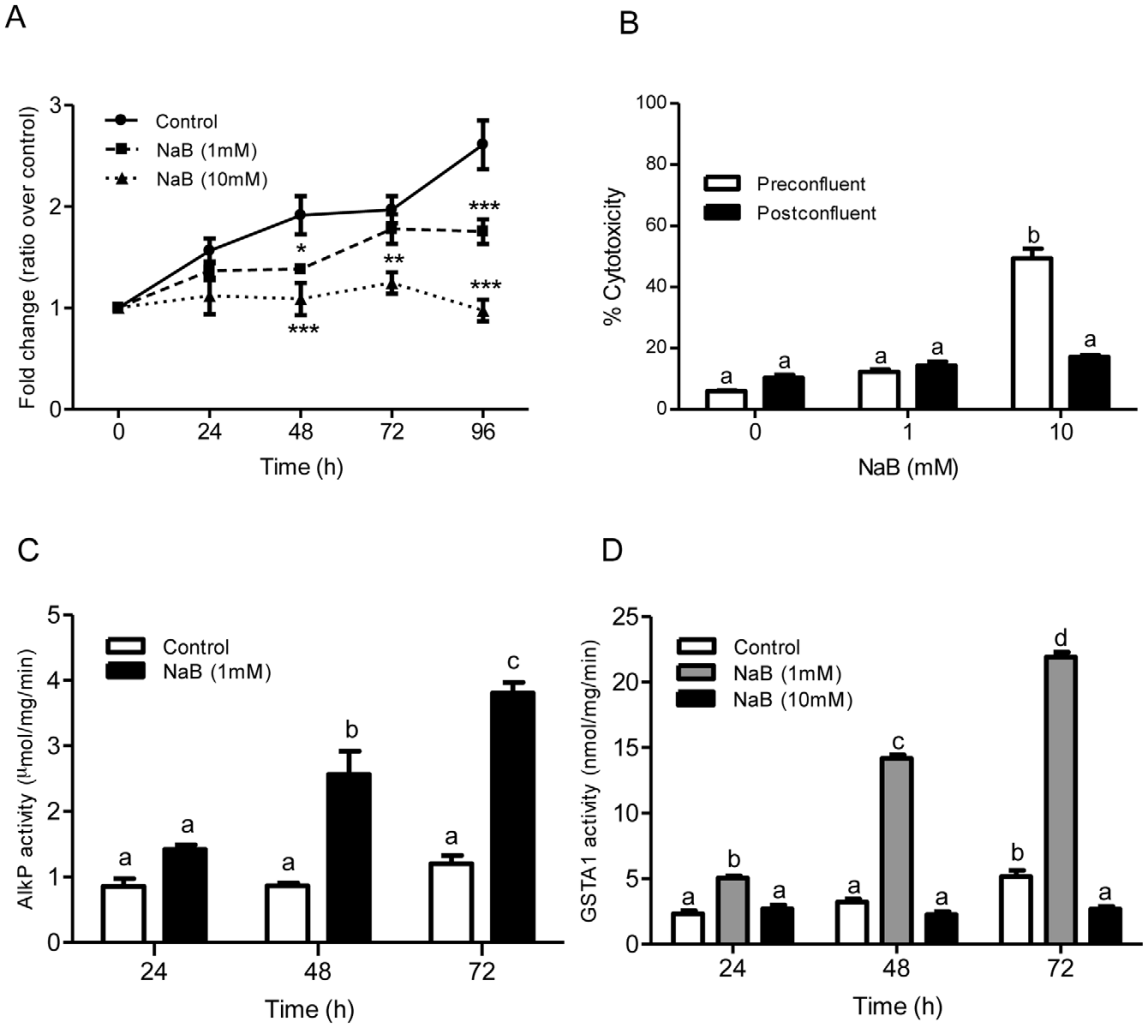
Distinct doses of NaB differently affect cell proliferation and AlkP and GSTA1 enzyme activities. Preconfluent Caco-2 cells were treated with NaB (1 mM and 10 mM) in serum-free media. (A) Cellular proliferation was assessed from 24–96 h. Asterisks depict significant differences between control and NaB treatments (*, p≤0.05; **, p≤0.01 and ***, p≤0.001). (B) Cytotoxicity was determined in preconfluent and postconfluent Caco-2 cells treated with 1 mM and 10 mM NaB at 48 h. Cytotoxicity measured LDH release and presented as % cytotoxicity. (C) AlkP activity (µmol/mg/min) and (D) GSTA1 activity (nmol/mg/min) was determined. Values represent the mean ± S.E. of three independent experiments with six replicates each. Bars indicated by different letters differ significantly from one another (p≤0.001).

Since GSTA1 modulation affected cellular proliferation and induced changes in cell cycle phase distribution, we further investigated the relationship between GSTA1 expression and transition through various cellular states in cells treated with NaB. Two concentrations of NaB that are known to cause either cellular differentiation (1 mM) or apoptosis (10 mM) were used. To determine the effect of NaB on cellular proliferation, a MTS assay was performed on preconfluent Caco-2 cells treated with NaB (1 and 10 mM) for up to 96 h (Fig. 5A). A concentration of 1 mM NaB caused a significant decrease in proliferation at 48 h (p<0.05) and at 96 h (p<0.001) compared to controls. A concentration of 10 mM NaB caused a greater reduction in cellular proliferation at 48, 72 and 96 h (p<0.001) compared to controls. We confirmed that reduction in proliferation by 1 mM NaB did not result in cytotoxicity whereas 10 mM NaB caused a significant increase in cytotoxicity in preconfluent cells (49.4%, p<0.001) and not in postconfluent cells (Fig. 5B).

In preconfluent Caco-2 cells treated with 1 mM NaB for 72 h, cellular differentiation was demonstrated by progressive increases in AlkP activity of 2.9-fold at 48 h (p<0.01) and 4.4-fold at 72 h (p<0.001) (Fig. 5C). No significant changes in AlkP activity were observed in untreated cells.

NaB differentially affected GSTA1 activity in Caco-2 cells treated with 1 or 10 mM NaB over a 72 h period (Fig. 5D). While GSTA1 activity significantly increased by 2.2-fold at 72 h in untreated control cells, a much greater progressive increase in GSTA1 activity (4.4-fold) occurred with 1 mM NaB treatment reaching a maximum activity of 21.9 nmol/mg/min at 72 h, (p<0.001). Conversely, at 72 h GSTA1 activity decreased from 5.2 nmol/mg/min in control cells to 2.7 nmol/mg/min with NaB (10 mM) treatment.

### Modulation of GSTA1 does not affect sodium butyrate-induced differentiation

**Figure 6:**
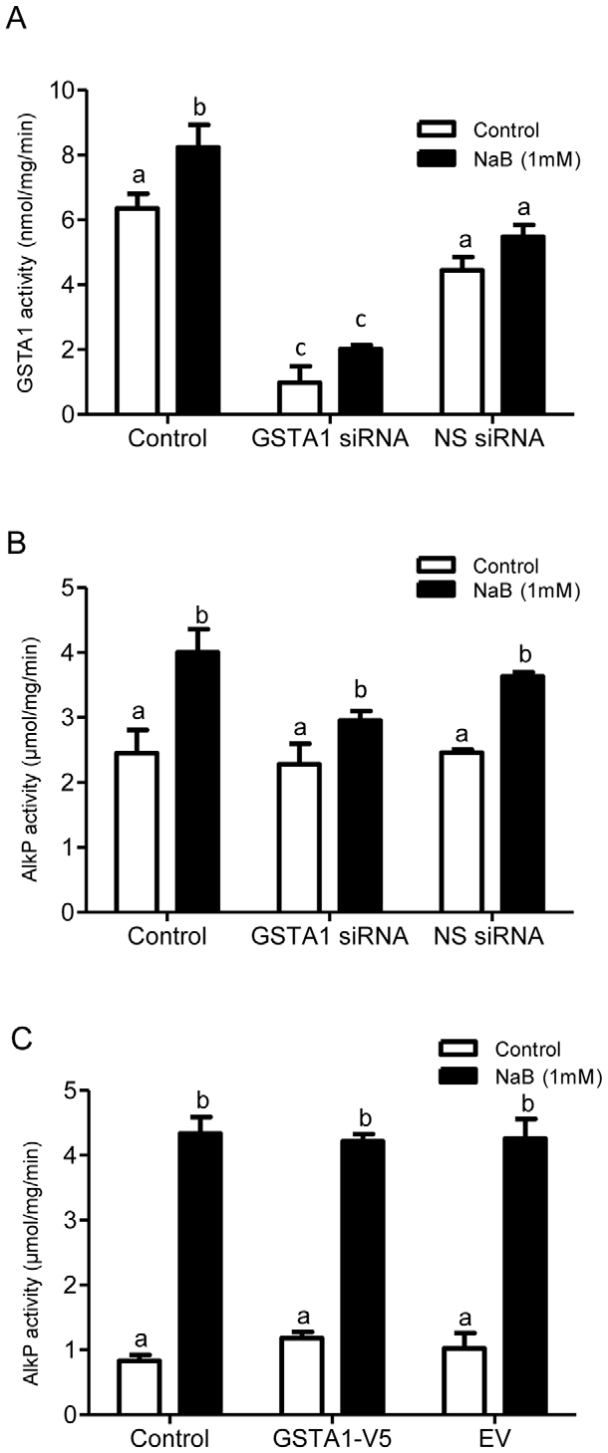
Modulation of GSTA1 does not affect NaB-induced differentiation. Preconfluent Caco-2 cells were transiently transfected with 40 nM of GSTA1 siRNA or non-specific (NS) siRNA and after 72 h, cells were treated with 1 mM NaB for 72 h; (A) GSTA1 activity (nmol/mg/min) was determined in GSTA1 down-regulated cells. (B) AlkP activity (µmol/mg/min) was measured to determine the effect of GSTA1 down-regulation on NaB-induced differentiation. (C) Preconfluent Caco-2 cells were transiently transfected with one µg of either GSTA1-V5 or empty vector (EV) for 48 h and were treated with 1 mM NaB for 48 h. AlkP activity (µmol/mg/min) was measured to determine the effect of GSTA1 over-expression on NaB-induced differentiation. Values represent the mean ± S.E. of three independent experiments with six replicates each. Bars indicated by different letters differ significantly from one another (p≤0.001).

The data thus far demonstrates that a low concentration of NaB (1 mM) induces differentiation, reduces cellular proliferation, and increases GSTA1 activity. To investigate whether GSTA1 plays a direct role in NaB-induced differentiation, endogenous GSTA1 was either knocked down or over-expressed in preconfluent Caco-2 cells. GSTA1 activity was determined in GSTA1-down-regulated cells with and without NaB (1 mM) treatment (Fig. 6A). NaB (1 mM) significantly increased GSTA1 activity at 72 h by 1.4-fold (p<0.05) in control cells. In cells transfected with GSTA1 siRNA, a 78% (p<0.001) and 63% (p<0.001) decrease in GSTA1 activity was observed with and without NaB (1 mM) treatment respectively as compared to cells transfected with non-specific (NS) siRNA. NaB did not alter GSTA1 activity in cells transfected with GSTA1 siRNA and non-specific siRNA (Fig. 6A).

AlkP activity was assessed in cells with GSTA1 knocked down with and without NaB treatment to determine the effect of GSTA1 knock-down on cellular differentiation (Fig. 6B). As expected, NaB caused a significant increase in AlkP activity from 2.4 µmol/mg/min to 4.0 µmol/mg/min (1.7-fold; p<0.001), 3.7 µmol/mg/min (1.4-fold; p<0.01) in nontransfected control cells and NS siRNA transfected cells treated with NaB (1 mM) respectively. However, AlkP activity in NaB-treated cells transfected with GSTA1 siRNA was not significantly different from controls.

To determine the effect of GSTA1 over-expression on differentiation, AlkP activity was determined in preconfluent Caco-2 cells transfected with the GSTA1-V5 expression plasmid (Fig. 6C). NaB caused a greater than 4-fold increase (p<0.001) in AlkP activity in all cells indicating that NaB-mediated increases in AlkP activity were independent of the level of GSTA1. Differences in AlkP activity in control cells in panels B and C are attributed the different passage numbers and stages of confluency of cells in GSTA1 overexpression and knockdown experiments at the time of harvesting.

To determine the role of GSTA1 on differentiation in the absence of NaB treatment, AlkP activity was assessed 15 days after knockdown of GSTA1 by transfection with GSTA1 siRNA. At day 15, there was no statistically significant difference in the increase in AlkP activity between controls (7 fold increase) and cells in which GSTA1 is knocked down (6.2 fold increase).

### Modulation of GSTA1 does not affect sodium butyrate-induced apoptosis

**Figure 7:**
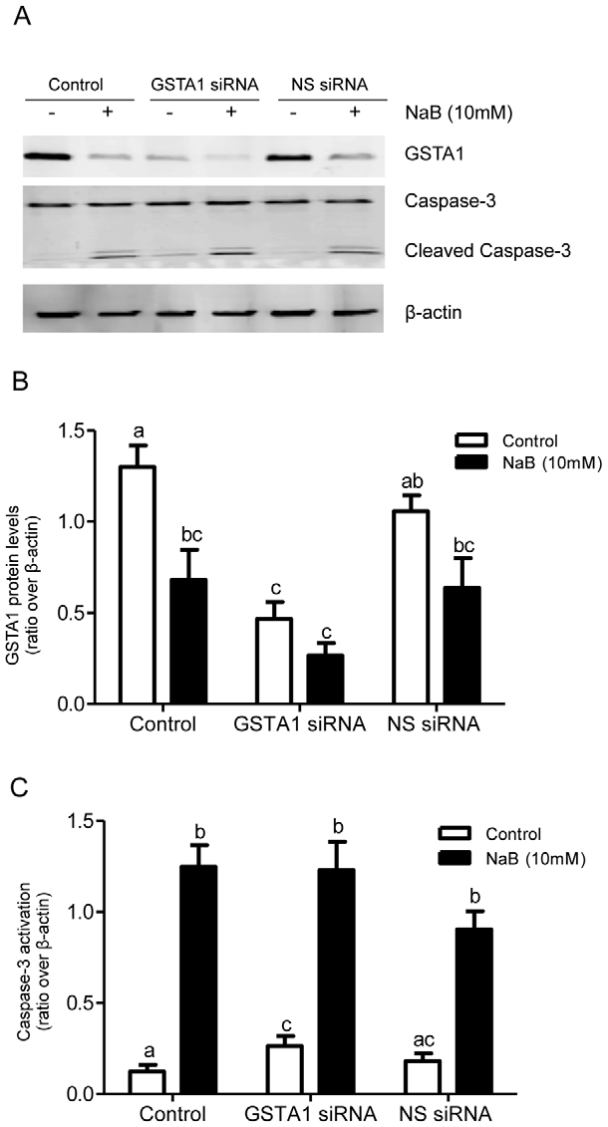
GSTA1 down-regulation does not affect the sensitivity of Caco-2 cells to NaB-induced apoptosis. (A) Representative western blots of GSTA1 (∼25 KDa), endogenous caspase-3 (∼35 KDa), activated caspase-3 (∼19 KDa and 17 KDa) in Caco-2 cells. Preconfluent Caco-2 cells were transiently transfected with 40 nM of GSTA1 siRNA or non-specific (NS) siRNA for 72 h and treated with NaB (10 mM) for 48 h. β-actin (∼42 KDa) was used as a protein loading control. Densitometric analysis of (B) GSTA1 levels and (C) caspase-3 activation in GSTA1 down-regulated cells with and without NaB (10 mM) treatment. Values represent the mean ± S.E of three independent experiments with three replicates each. Bars indicated by different letters differ significantly from one another (p≤0.05).

To investigate the possibility that GSTA1 down-regulation is required to increase the sensitivity of Caco-2 cells to apoptosis induced by 10 mM NaB, endogenous GSTA1 was transiently knocked down in preconfluent Caco-2 cells using siRNA technology. Endogenous GSTA1 protein levels significantly decreased by 65% (p<0.001) following transfection with GSTA1 specific-siRNA compared to transfection with non-specific siRNA (Figure 7A and B). Apoptosis was assessed by caspase-3 activation in GSTA1 down-regulated cells treated with 10 mM NaB. Immunoblot analysis shows that NaB (10 mM) resulted in increases of caspase-3 activity of 10.1, 9.9- and 7.3-fold in control cells and those transfected with GSTA1 siRNA and NS siRNA respectively (p<0.001). There was no significant difference in caspase-3 activation in NaB-treated controls and transfected cells (Fig. 7A and C).

**Figure 8:**
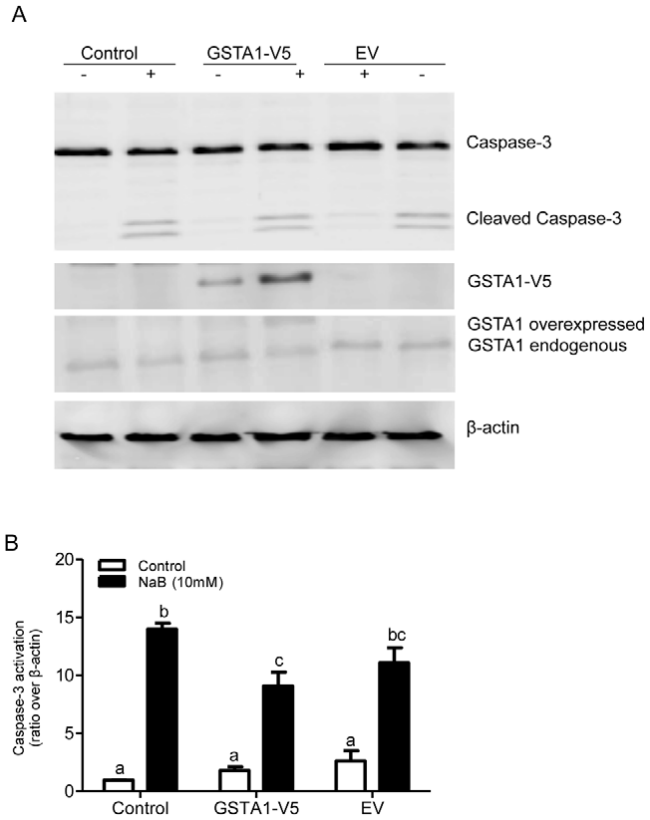
GSTA1 over-expression does not interfere with NaB-induced apoptosis. (A) Representative western blots of V5 (∼26 KDa), endogenous caspase-3 (∼35 KDa) and activated caspase-3 (∼19 KDa and 17 KDa) in Caco-2 cells. Preconfluent Caco-2 cells were transiently transfected with one µg of either GSTA1-V5 or empty vector (EV) for 48 h and treated with NaB (10 mM) for 48 h. β-actin (∼42 KDa) was used as a protein loading control. (B) Densitometric analysis of caspase-3 activation in GSTA1 over-expressed cells with and without NaB (10 mM) treatment. Values represent the mean ± S.E of three independent experiments. Bars indicated by different letters differ significantly from one another (p≤0.001).

We also assessed caspase-3 activation in GSTA1 over-expressing cells treated with NaB (10 mM). Immunoblot analysis shows that caspase-3 activation occurred in all cells treated with NaB (10 mM). NaB-mediated increases in caspase-3 activation were approximately 13.9-, 9.1- and 11.1-fold in control cells and those transfected with GSTA1-V5 and the empty vector respectively (p<0.001). There was no significant difference in the degree of caspase-3 activation in NaB-treated GSTA1-V5 and empty vector transfected cells (Fig. 8A and B).

### NaB (10 mM) causes GSTA1-JNK complex dissociation without activating JNK in Caco-2 cells

**Figure 9:**
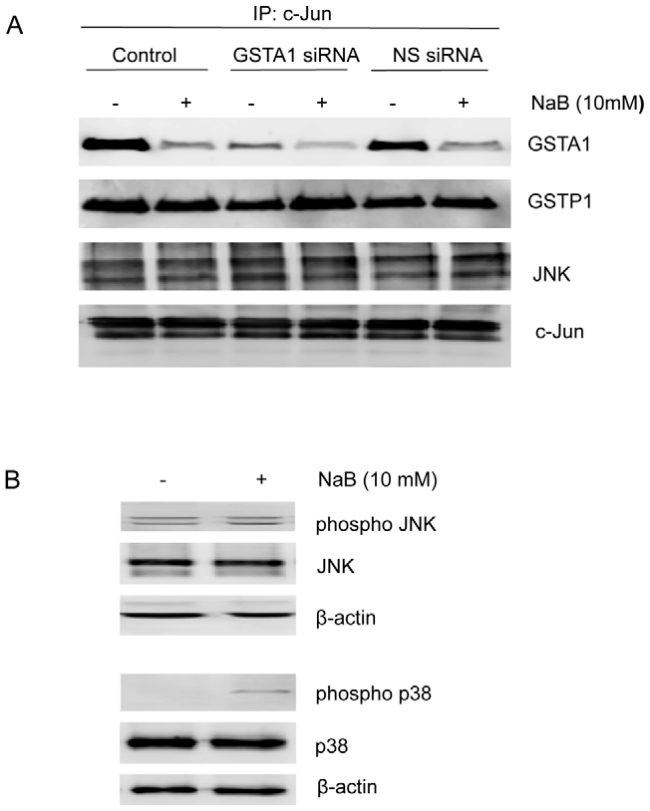
NaB (10 mM) causes GSTA1-JNK complex dissociation without activating JNK in Caco-2 cells. (A) Representative western blot of GSTA1 (∼25 KDa) and GST Pi (∼26 KDa) protein levels in GSTA1-JNK complexes. Cells were transiently transfected with GSTA1 siRNA and non-specific (NS) siRNA for 72 h and treated with 10 mM NaB. GSTA1-JNK complexes were then pulled-down from cell lysates using c-Jun fusion beads. (B) Representative western blot of phosphorylated JNK (∼54 KDa and 46 KDa) and phosphorylated p38 (∼43 KDa) protein expression in preconfluent Caco-2 cells with the treatment of 10 mM NaB. β-actin (∼42 KDa) was used as a protein loading control.

We hypothesized that apoptosis caused by 10 mM NaB is also associated with dissociation of GSTA1-JNK complexes. The effect of NaB (10 mM) on GSTA1-JNK complex integrity was determined in cells in which GSTA1 knocked down by siRNA as well as in control cells and in cells transfected with non-specific siRNA (Fig. 9A). GSTA1-JNK complexes were pulled-down using c-Jun fusion protein beads and GSTA1 levels were determined by western blot analysis. Knock-down of GSTA1 reduced levels of GSTA1 proteins in complexes by approximately 75%. NaB (10 mM) caused dissociation of the GSTA1-JNK complexes at 72 h in control and transfected cells (Fig. 9A). There was no difference in the level of GSTP1 protein complexed with JNK in NaB-treated and untreated controls. While there was no difference in JNK activation, as measured by phosphorylated JNK levels, in NaB-treated and untreated controls, phosphorylated p38 levels increased following treatment with 10 mM NaB (Fig. 9B).

## Discussion

The objective of this study was to determine if GSTA1 plays a direct role in modulating cellular proliferation, differentiation and apoptosis in Caco-2 cells. In view of the role of GSTA1 in controlling cellular stress signaling via JNK inhibition [14], we postulated that expression of GSTA1 may modulate transitioning through various cellular states. We investigated this concept by examining the influence of direct manipulation of GSTA1 expression (i.e. knock-down and over-expression) in modulating NaB-mediated transitioning through proliferation to differentiation to apoptosis. We also examined GSTA1 expression in Caco-2 cells following exposure to different concentrations of NaB, a short chain fatty acid, that induces differentiation and apoptosis in colon cancer cell lines [18]. A clearer understanding of the role of GSTA1 expression in modulation of transitioning between cellular states has important implications in diseases such as cancer in which there is an imbalance in cellular proliferation, differentiation and apoptosis.

Our results indicate that GSTA1 expression influences the proliferative status of Caco-2 cells, such that low GSTA1 expression provides cellular conditions that are conducive to enhanced proliferation. The evidence is as follows: i) GSTA1 expression in preconfluent cells is low compared to the higher levels observed in differentiated postconfluent cells, ii) NaB at a concentration of 1 mM increases GSTA1 activity, suppresses Caco-2 cell proliferation in MTS assays and induces a differentiated phenotype, iii) overexpression of GSTA1 suppresses proliferation in Caco-2 cells transfected with a GSTA1 pcDNA 3.1/V5-His TOPO vector, iv) suppression of GSTA1 expression in Caco-2 cells transfected with GSTA1 siRNA increases the percentage of cells in S phase as determined by flow cytometry as well as the overall proliferative rate in MTS assays. Previous studies have shown that GSTA1 over-expression in cell lines with no detectable GSTA1 levels such as the human retinal pigment epithelial (RPE) cells and human lung cancer (H69) cells does not affect growth rate [24], [25]. However, in both studies data was not presented to support the claim that overexpression of hGSTA1-1 did not alter growth kinetics and details regarding the timeframe over which cell growth was assessed was not clearly indicated. In the current study, the most profound reduction in cell growth due to GSTA1 overexpression was observed at 72 h suggesting that the assessment of GSTA1-1 effects on the proliferation of RPE and H69 cells may have occurred too early. Other studies have shown both *in vivo* and *in vitro* that GST Pi influences cellular proliferation [8], [26], [27]. Ruscoe et al., (2001) demonstrated that mouse embryo fibroblasts, isolated from GSTP1-1 knock-down mice (GSTPi ^−/−^), doubled at a faster rate compared to the cells from GSTPi ^+/+^ wild-type mice [26]. Their results indicated a mechanism involving GSTP1-1-mediated control of cellular mitogenic pathways including signalling kinases JNK1 and ERK1/ERK2 that influence proliferation. Another study demonstrated differential effects of GSTP1 on cell proliferation dependent on haplotype with GSTP1*A reducing cellular proliferation and GSTP1* C allele having no effect in NIH3T3 fibroblasts [8]. In contrast, Hokaiwado (2008) demonstrated that GSTPi knock down using siRNA resulted in significant decrease in proliferation rate of human prostate cancer PC3 cells [27]. While the influence of GSTPi on Caco-2 cell proliferation was not directly examined in the current study the results clearly demonstrate that GSTPi expression does not change in differentiating Caco-2 cells in which GSTA1 is knocked down or following NaB treatment. This suggests that the influence of different GST isozymes on cellular proliferation may be cell line-dependent.

Postconfluent Caco-2 cells differentiate and acquire a mature phenotype with increased expression of alkaline phosphatase, villin, E-cadherin and dipeptidyl peptidase-4 [28], [29]. More relevant to our study is the marked up-regulation of GSTA1 expression during differentiation of postconfluent Caco-2 cells [14], [30], [31]. This finding supports the results of a study by Scharmach et al., 2009 that demonstrated marked induction of GSTA1, GSTA2 and to a lesser extent GSTA3 in association with Caco-2 cell differentiation whereas constant high expression was observed for GSTP1 and GSTO1, marginal expression for GSTA4, GSTT2 and GSTZ1 and no expression of GSTM5 and GSTT1 [30]. The same study demonstrated GSTA1 induction in proliferating cells exposed to 5 mM NaB for 24 h. Because of the observed upregulation of GSTA1 with increased confluency, we speculated that a causal relationship might exist between expression of this isoenzyme and differentiation of Caco-2 cells. Moreover, the finding that NaB induces GSTA1 at a concentration (1 mM) that suppresses proliferation and promotes Caco-2 cell differentiation, also suggests an association of GSTA1 with a differentiated phenotype. However, our overexpression and knockdown experiments designed to directly assess a causal relationship did not support the hypothesis that GSTA1 expression at high levels was a requisite condition for cellular differentiation. Previous studies have shown that 1 mM NaB induces AlkP activity, and hence differentiation, in both Caco-2 and HT-29 cells [18]. Others have demonstrated that NaB induced total GST activity in HT-29 cells including the isoforms GSTA1/2, GSTM2 and GSTP1 [32]. It is possible that GSTA1 induction may occur as a cytoprotective response to increased oxidative stress. For example, previous studies have shown that GSTA1-1 overexpression attenuates H_2_O_2_-induced oxidative stress and protects RPE and H69 cells against the associated cytotoxicity likely by attenuating lipid peroxidation [24], [25].

Interestingly, different concentrations of NaB also had differential effects on *GSTA1* expression in Caco-2 cells. As mentioned above, while 1 mM NaB induces differentiation and increases GSTA1 activity, 10 mM NaB decreases GSTA1 activity, activates caspase-3 activity and causes a complete cessation of proliferation as observed by MTS assays. We directly examined the role of GSTA1 in NaB-mediated apoptosis using the same approach of over-expressing and knocking down GSTA1 in preconfluent Caco-2 cells using GSTA1-V5 overexpression and siRNA technology respectively. Our data indicate that direct modulation of GSTA1 expression does not alter NaB-induced apoptosis in Caco-2 cells. However, Louis et al., (2004) demonstrated in breast cancer MCF-7 cells that NaB-induced apoptosis was associated with a pronounced depletion of intracellular glutathione levels and induction of antioxidant enzymes including glutathione reductase, glutathione peroxidase and catalase [33]. Moreover, Matthews et al 2012, demonstrated that butyrate-mediated apoptosis is associated with reductions in glutathione availability and increases in levels of reactive oxygen species in Caco-2 cells [34]. We were unable to demonstrate a change in intracellular glutathione levels in Caco-2 cells following NaB-induced apoptosis (data not shown).

We have previously demonstrated that GSTA1 forms complexes with JNK in Caco-2 cells and that over-expression of GSTA1 increases resistance to complex dissociation and oxidative stress-induced apoptosis [14]. Our current results show that GSTA1 down-regulation reduces the degree of GSTA1-JNK complex formation and that 10 mM NaB causes these complexes to dissociate. However, the relevance of these results is minimized in view of the fact that 10 mM NaB did not increase phosphorylation of JNK irrespective of the degree of GSTA1-JNK complex formation. Some GST isoforms are associated with signaling kinases and control cell proliferation and cell death by modulating the MAPK pathway and GST-specific inhibitors activate JNK and induce apoptosis [14], [35], [36], [37], [38]. Moreover, JNK is a key regulator in the pathway of programmed cell death [39]. Since we observed decreased GSTA1 activity and induction of apoptosis with NaB treatment, JNK activation was expected. NaB triggers apoptosis by activating the JNK/AP1 pathway and eventually transcriptional stimulation of Bax in human DiFi and FET colorectal carcinoma cells [40]. Nonetheless, we did not observe JNK activation with NaB-induced apoptosis in Caco-2 cells. Others have shown that treatment of Caco-2 and HT-29 cells with NaB increases p38 kinase activity which subsequently stimulates apoptotic pathways suggesting that other stress kinase pathways may be involved [41], [42]. This is supported by our finding that 10 mM NaB increases p38 phosphorylation in Caco-2 cells.

The findings of this study elucidate the involvement of GSTA1 in modulating cellular proliferation and NaB-induced differentiation and apoptosis. The results provide support for the hypothesis that low levels of GSTA1 are a requisite condition for Caco-2 cells to proliferate. While levels of GSTA1 are differentially modified by concentrations of NaB that stimulate cellular differentiation, or apoptosis, direct modulation of GSTA1 levels does not alter transitioning through these cellular states. The clinical implication of altering GSTA1 expression to control excessive cellular proliferation requires further investigation.
